# Editorial

**DOI:** 10.1007/s11609-020-00415-5

**Published:** 2020-10-26

**Authors:** Benjamin Seyd

**Affiliations:** Friedrich-Schiller-Universität Jena, Jena, Deutschland

Die Zyklen wissenschaftlichen Publizierens sind dem zielgenauen Timing von Interventionen in aktuelle Debatten üblicherweise nicht dienlich. Das gilt insbesondere dort, wo die Qualität solcher Interventionen – wie beim Berliner Journal für Soziologie – durch ein aufwändiges Peer-Review-Verfahren gewährleistet wird, Autor_innen es also vor Veröffentlichung noch mit Gutachterhinweisen, Herausgebervoten, Überarbeitungsrunden und gründlicher Redaktionsarbeit zu tun bekommen. Doch sind, wie vielerorts im Leben, glückliche Fügungen nicht auszuschließen. Und weil es um Soziologie geht, die stets mit (kleinen oder großen) gesellschaftlichen Problemen anfängt – und im Idealfall *etwas* damit anfängt –, bedeuten solche Fügungen meistens: Glück im Unglück.

Ein solches Glück im Unglück ist für uns nun auch der vorliegende Schwerpunkt zum Thema Solidarität. Als wir das Projekt auf Initiative von Annette Schnabel und Ulf Tranow in Angriff nahmen, war von Corona noch nichts zu spüren. Als die ersten Texte geschrieben waren und in die Begutachtung gingen, waren die beherrschenden Themen nicht Ansteckungszahlen, Lockdown, Hygienepläne und Versammlungsbeschränkungen. Stattdessen wirkte die „Flüchtlingskrise“ von 2015 nach, und in öffentlichen Debatten ging es zumeist nicht um den Schutz vor Ansteckung und möglichen staatlichen Übereifer, sondern um den Schutz des Klimas und staatliche Untätigkeit. Zuweilen merkt man den Beiträgen des Heftes diesen Hintergrund durchaus an; und dennoch haben sie – das ist das Glück im unbestreitbaren Unglück der multiplen Corona-Krise – nichts von ihrer Aktualität verloren.

Vielmehr ist Solidarität in Zeiten der Pandemie geradezu zum geflügelten Wort geworden. Ob die unter Quarantäne stehende Nachbarin versorgt werden muss, ein Laptop für das Homeschooling benötigt wird, das Stammcafé von Insolvenz bedroht oder das Toilettenpapier alle ist – wo immer Routinen unterbrochen sind und spontane Hilfe improvisiert werden muss, wird dieser Tage die verbindende Kraft der Solidarität beschworen. Man mag darin einen Indikator für die dominanten Hoffnungen in den flexiblen, mobilen, liquiden Verhältnissen sehen, unter denen Menschen im 21. Jahrhundert leben. Doch steigt mit überschwänglichen Bindungs- und Beistandshoffnungen auch die Gefahr von Enttäuschungen. Wie eine Kollegin, die Chefredakteurin der Immobilienzeitung, Brigitte Mallmann-Bansa, ihrerseits in einem Editorial beklagt:

Zusammenhalt. Rücksichtnahme. Hilfsbereitschaft. Die Corona-Zeit wird unsere Gesellschaft zu einer besseren machen, weil es eine nie gekannte Solidarität untereinander gibt. Das war die Hoffnung im Frühjahr. Davon ist nur wenig geblieben. In Wirklichkeit hat das Coronavirus die Menschen gespalten. In Kurzarbeiter und Überarbeitete, in Virusleugner und in Maßnahmen-Verfechter, in Ängstliche und Abgebrühte, in Corona-Gewinner und Corona-Verlierer. Wer vorher abgehängt war, der ist es jetzt umso mehr.

Der Immobilienwirtschaft erging es ebenso. Zwischen den unterschiedlichen Assetklassen und Nutzungsarten tut sich eine Kluft auf.^1^

Ernüchterungen wie dieser begegnet man am besten durch eine Rückkehr zum Ausgangspunkt. Es gilt, die eigenen Prämissen zu überprüfen: Was ist gemeint, wenn landauf, landab der Ruf nach Solidarität erschallt? Was können wir von ihr erhoffen, hätte sie unsere Erwartungen erfüllen können? Oder sind die Enttäuschungen mehr als nur ein Zufall, gibt es eine verborgene Kehrseite der Solidarität?

In diesem Sinne leisten die vorliegenden Beiträge zum Solidaritätsschwerpunkt konzeptuelle und empirische Grundlagenarbeit.

Wenn Thilo Fehmel in seinem theoretischen Beitrag zwischen Konflikten *um *und Konflikten *statt* Solidarität unterscheidet, wenn Doris Bühler-Niederberger in ihrem materialgesättigten Aufsatz die Variationsbreite familialer Solidarität erkundet, wenn Annette Schnabel in ihrer explorativen Studie dem Zusammenhang zwischen wohlfahrtsstaatlich und gemeinschaftlich organisierter Solidarität nachspürt – dann geht es stets darum, wie Annette Schnabel und Ulf Tranow in ihrer systematisierenden Einführung (die die einzelnen Beiträge ausführlicher vorstellt) schreiben, „nicht nur die inklusive und vergemeinschaftende Seite von Solidarität“ in den Blick zu nehmen, sondern auch die unweigerlich damit einhergehenden „Grenzziehungen samt ihrer Folgewirkungen, durch die wechselseitige Unterstützungs- und Verantwortungspraxen erst ermöglicht werden“. Und wenn Stephan Lessenich sich in seinem Artikel der dunklen Kehrseite wohlfahrtsstaatlich institutionalisierter Solidarität zuwendet und Jörg Althammer und Maximilian Sommer in ihrem Essay die Potenziale einer von moralischer Emphase entlasteten „strukturellen Solidarität“ eruieren, dann gilt ebenso: Zu einem gehaltvollen analytischen Verständnis von Solidarität beizutragen, indem ihren Grenzen, d. h. ihren Ein- und Ausschlüssen von Personen und Leistungen sowie den Konflikten, die sich daran entzünden, nachgegangen wird – das ist die Idee dieses Schwerpunkts.

Eine solche wirklichkeitswissenschaftliche Klärung entspricht ganz dem Anspruch des Berliner Journals. Nicht auszuschließen ist, dass dem geflügelten Wort „Solidarität“ im Zuge dieser Auseinandersetzung die Flügel etwas gestutzt werden und manche allzu hochfliegende Hoffnung auf den Boden der Tatsachen zurückgeholt wird. Doch ein geerdeter Blick auf die komplizierten Verhältnisse ist in diesen Zeiten ja nicht das Schlechteste.

## Footnotes

1 https://redaktion.iz.de/a.php?sid=2zmix.2ca3qtl,f=1,u=845f9d14e26c0fcfbe447b059eb7d646,n=2zmix.2ca3qtl&utm_source=newsletter&utm_medium=e-mail&utm_campaign=Wochennewsletter%20kw38_2020,l=84lnib.2mj2h2l. Zugegriffen: Sept. 2020.

